# Supplementary material for: Structural mechanisms of TRPM7 activation and inhibition
Source: Nat Commun. 2023 May 8;14:2639. doi: 10.1038/s41467-023-38362-3 (PMC10167348; doi:10.1038/s41467-023-38362-3)
Supplement: Supplementary file 1 — Supplementary Information [file 41467_2023_38362_MOESM1_ESM.pdf]

Supplementary Information

Structural mechanisms of TRPM7 activation and inhibition

Kirill D. Nadezhdin[1], Leonor Correia[2], Chamali Narangoda[3], Dhilon S. Patel[3], Arthur Neuberger[1], Thomas Gudermann[2,4], Maria G. Kurnikova[3,*], Vladimir Chubanov[2,*], and Alexander I. Sobolevsky[1,*]

[1]Department of Biochemistry and Molecular Biophysics, Columbia University, New York, NY, USA

[2]Walther-Straub Institute of Pharmacology and Toxicology, LMU Munich, Munich, Germany

[3]Chemistry Department, Carnegie Mellon University, Pittsburgh, PA, USA

[4]Comprehensive Pneumology Center, German Center for Lung Research (DZL), Munich, Germany

[*]Corresponding authors: M.G.K. (kurnikova@cmu.edu), V.C. (vladimir.chubanov@lrz.uni-muenchen.de) or A.I.S. (as4005@cumc.columbia.edu)

This PDF file includes:

Supplementary Figures 1-10

Supplementary Tables 1-4

Reference

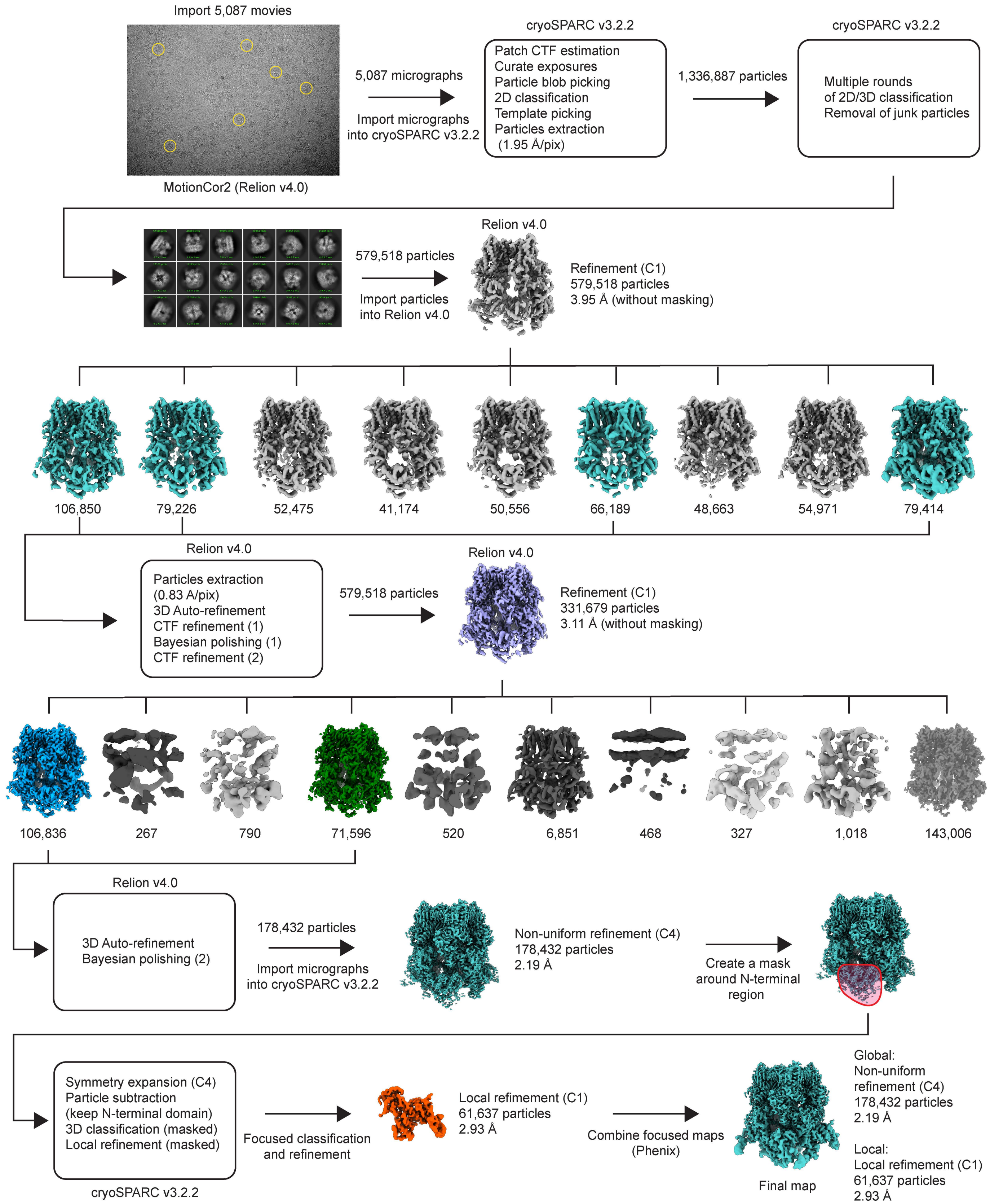

Supplementary Fig. 1 | Cryo-EM data processing workflow. The cryo-EM processing workflow is illustrated for the 2.19-Å resolution $\text{TRPM7}_{\text{Closed}}$ structure.

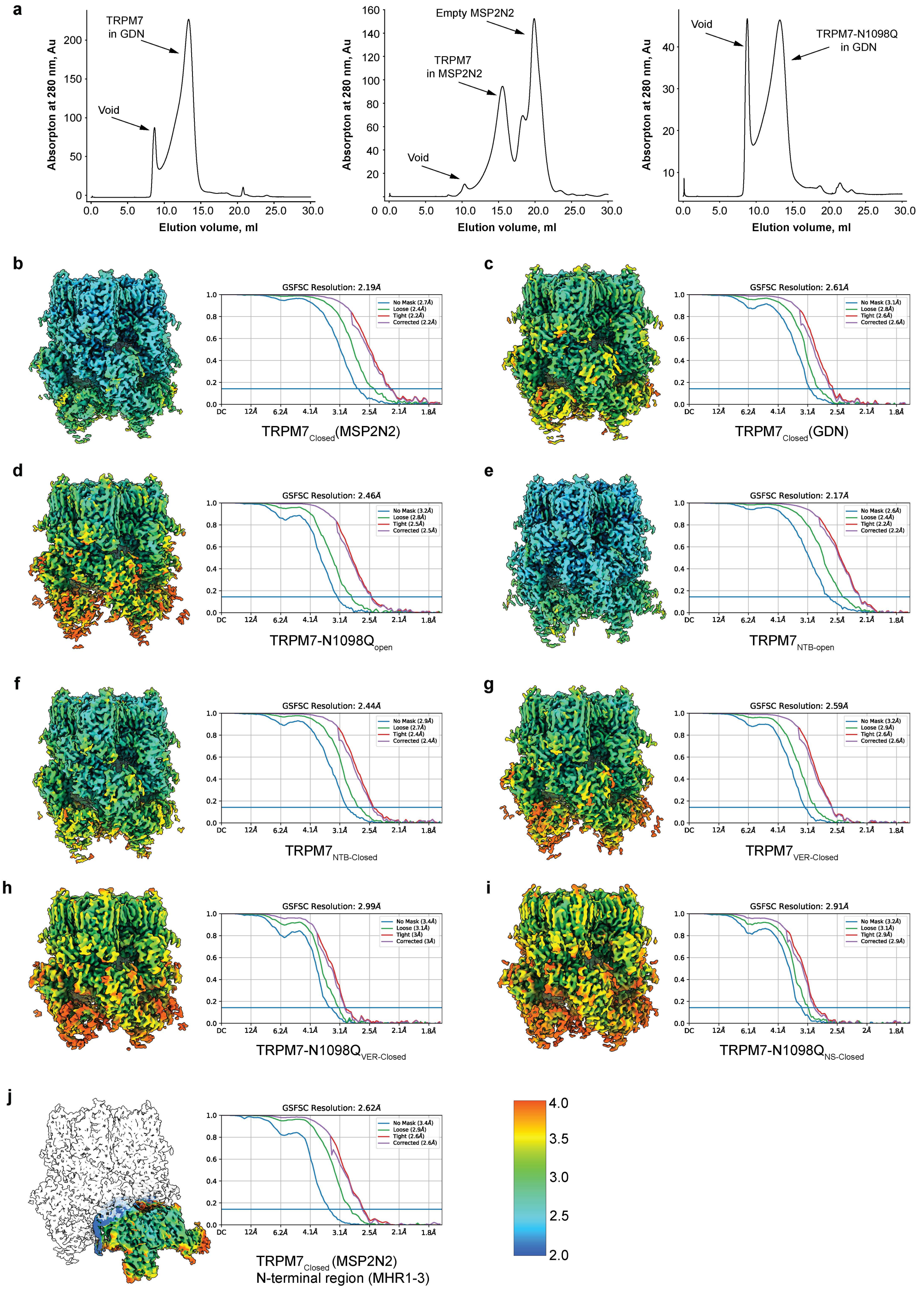

Supplementary Fig. 2 | Characteristics of Cryo-EM reconstructions. a SEC profiles for TRPM7 purified in 0.01% GDN (left), TRPM7 reconstituted in MSP2N2 nanodiscs (middle), and TRPM7-N1098Q purified in 0.01% GDN (right). **b-j** Cryo-EM maps colored according to the local resolution estimation in cryoSPARC[61] (left) and FSC curves calculated between half maps, with the overall resolution estimated using the FSC = 0.143 criterion[80] (right).

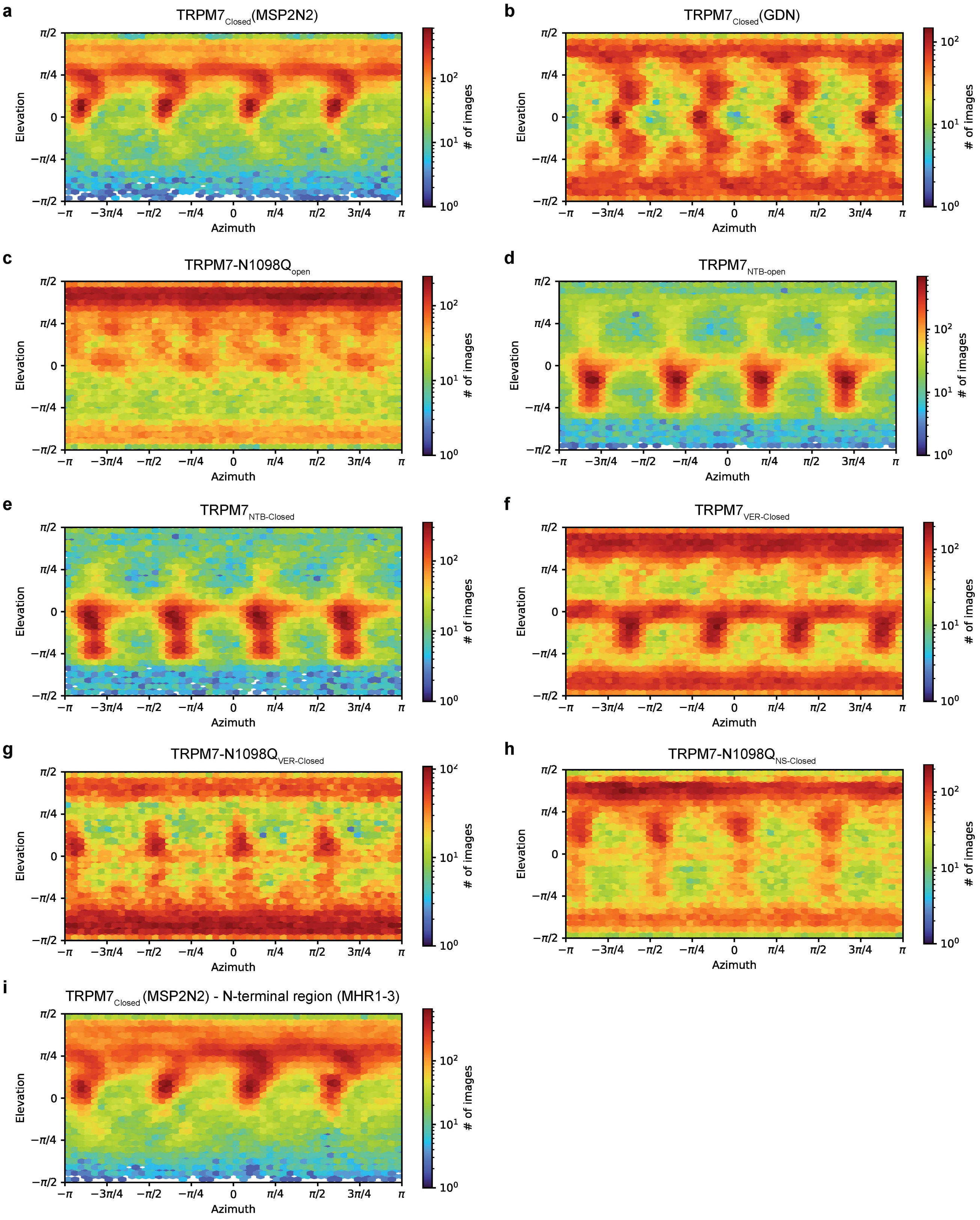

Supplementary Fig. 3 | Distribution of particle orientations contributing to the final cryo-EM reconstructions. a-i Angular distribution of particles calculated by cryoSPARC 3D Refinement reconstruction algorithm for TRPM7$_{closed}$ in MSP2N2 nanodiscs (**a**), TRPM7$_{closed}$ in GDN (**b**), TRPM7-N1098Q$_{open}$ (**c**), TRPM7$_{NTB-open}$ (**d**), TRPM7$_{NTB-closed}$ (**e**), TRPM7$_{VER-closed}$ (**f**), TRPM7-N1098Q$_{VER-closed}$ (**g**), TRPM7-N1098Q$_{NS-closed}$ (**h**), and TRPM7 MHR1-3 domain (**i**).

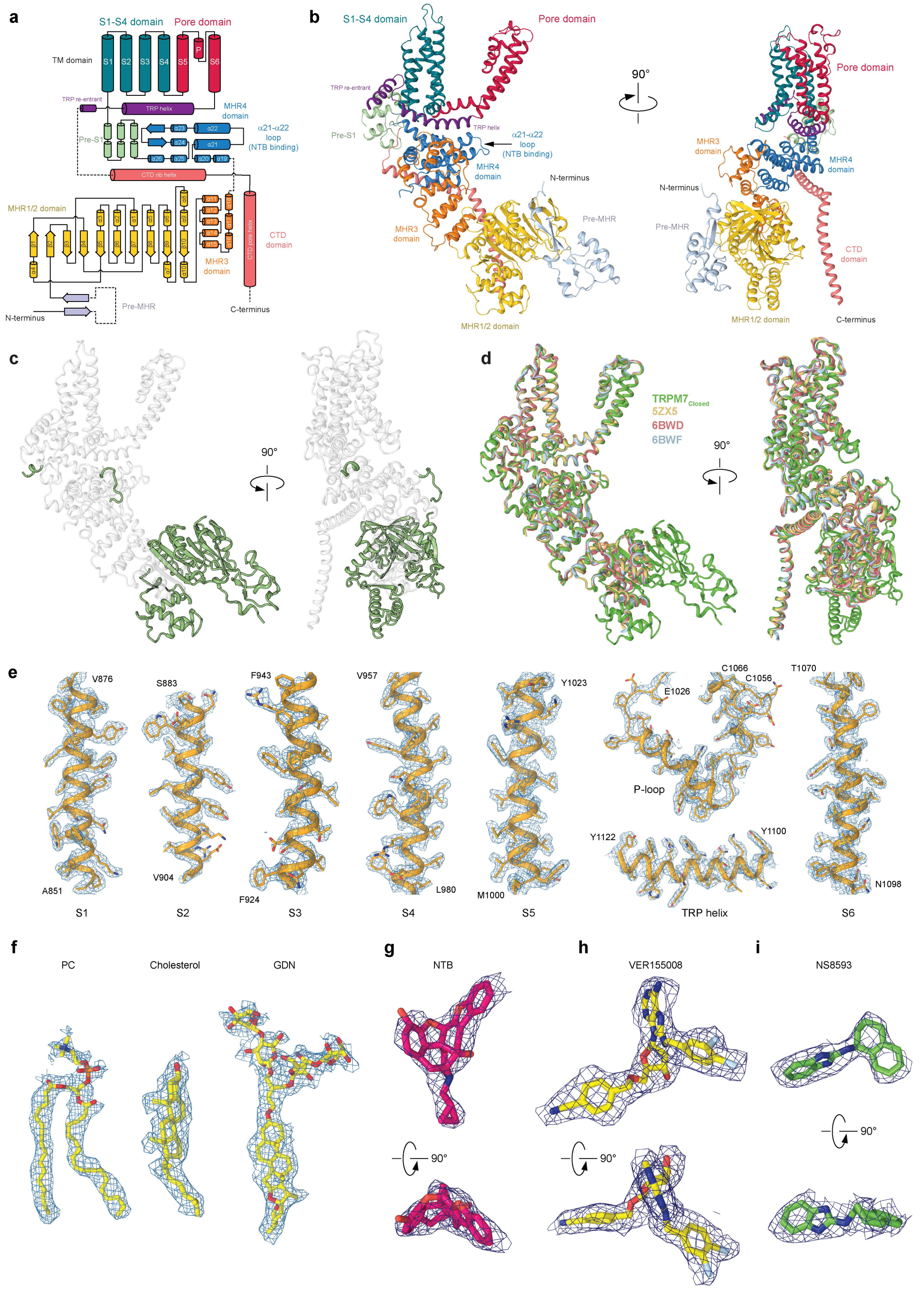

Supplementary Fig. 4 | Overall architecture of the TRPM7 subunit, comparison with previously published structures and examples of cryo-EM density. a TRPM7 subunit topology diagram colored according to domain organization. **b** Cartoon representation of TRPM7 subunit colored according to the domain organization. **c** *De novo* built structural elements in the single subunit of $TRPM7_{Closed}$ (green), with the previously resolved regions (PDB IDs: 5ZX5) shown in grey. **d** Structural alignment of a single subunit from $TRPM7_{Closed}$ in nanodisc (green) and published structures in detergent (yellow, PDB ID: 5ZX5; red, PDB ID: 6BWD; blue, PDB ID: 6BWF)[42]. **e** Fragments of the $TRPM7_{Closed}$ TMD with the structural model shown as a ribbon and sticks and the corresponding cryo-EM density as a blue mesh. **f** Representative densities (blue mesh) for lipids phosphatidylcholine (PC), cholesterol, and detergent glycol-diosgenin (GDN) shown in sticks (yellow). **g-i** Representative densities (blue mesh) for TRPM7 ligands naltriben (NTB) (**g**), VER155008 (**h**), and NS8593 (**i**) shown in sticks (pink, yellow and green, respectively).

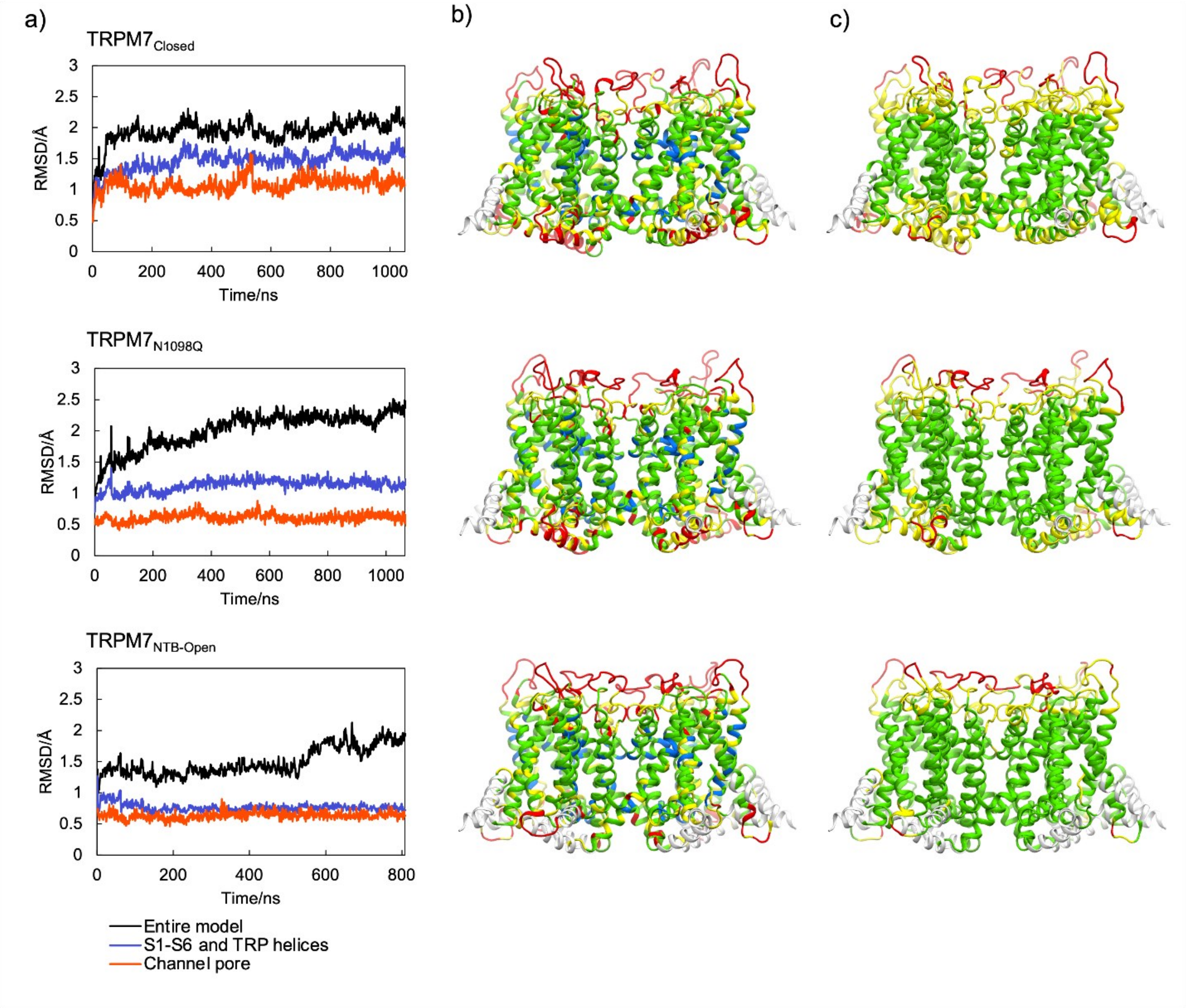

Supplementary Fig. 5 | Protein stability during MD simulations. a Root-mean-square deviation (r.m.s.d) from the initial structures in the production runs of representative systems of $TRPM7_{closed}$, TRPM7-N1098Q, and $TRPM7_{NTB-open}$. For each system, Cα r.m.s.d of the entire model (black), S1-S6 and TRP helices (blue), and channel pore (orange) are shown. See **Supplementary Table 2** for the simulation information. **b** Average r.m.s.d per residue for representative systems. The backbone is colored by RMSD (blue: r.m.s.d < 1 Å, green: 1.0 Å ≤ r.m.s.d < 2.0 Å, yellow: 2.0 Å ≤ r.m.s.d <3.0 Å, red: r.m.s.d ≥ 3.0 Å). Protein residues that were positionally restrained in the simulation are colored in white. **c** Root-mean-square fluctuation (r.m.s.f.) per residue for representative systems. The backbone is colored by r.m.s.f. (green: r.m.s.f. < 1.0 Å, yellow: 1.0 Å ≤ r.m.s.f. < 2.0 Å, red: r.m.s.f. ≥ 2.0 Å). Protein residues that were positionally restrained in the simulation are colored in white.

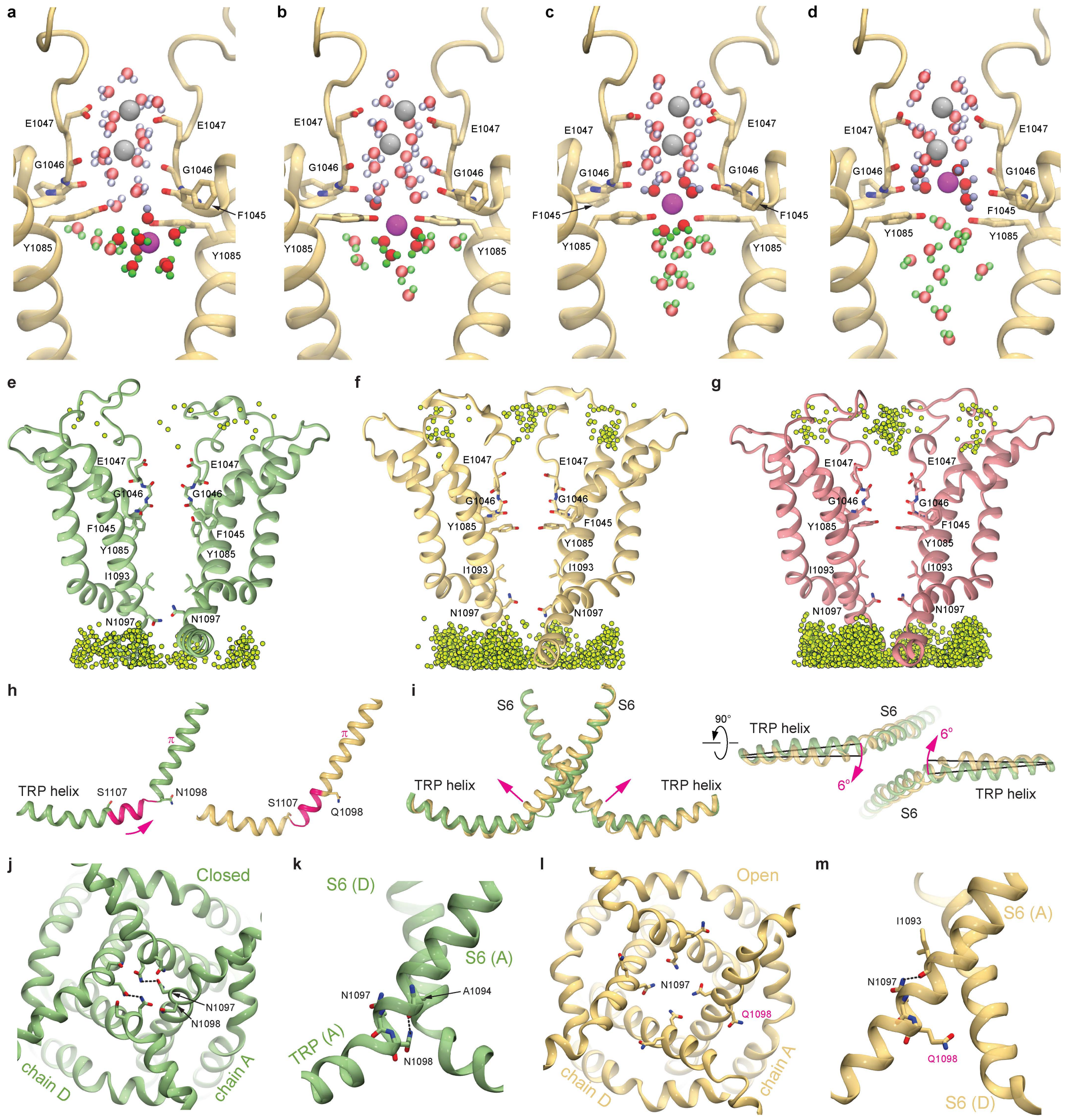

Supplementary Fig. 6 | Role of select residues in TRPM7 ion permeation and gating highlighted by MD simulations. a-d K^+ ion position during its permeation through TRPM7-N1098Q$_{open}$ channel and interaction with the Y1085 side chain before (**a-b**) and after (**c-d**) entering the selectivity filter (E1047, G1046). K^+ ions are shown as pink and grey balls. Water molecules initially located above and below Y1085 residues are shown as red spheres with differently colored hydrogens. Water does not cross the constriction formed by the Y1085 residues during ion permeation in the majority of simulations. **e-g** Cumulative distribution of Cl^- ions (green spheres) in the MD simulations of TRPM7$_{Closed}$ channel with no applied voltage (**e**), TRPM7-N1098Q$_{open}$ channel under applied voltage (**f**), and TRPM7$_{NTB\text{-}open}$ channel under applied voltage (**g**). **h** S6 and TRP helix, with the region undergoing the two helical-turn exchange during gating highlighted in pink. The pink arrow shows the direction of exchange between TRPM7$_{closed}$ and TRPM7-N1098Q$_{open}$ structures. **i** Superposition of S6 and TRP helices in two diagonal subunits of TRPM7$_{closed}$ (green) and TRPM7-N1098Q$_{open}$ (yellow), with the other two subunits removed for clarity. The pink arrows indicate structural rearrangements. **j-k** Close-up views of the TRPM7$_{closed}$ channel intracellular entrance (**j**) and S6-TRP helix regions in the neighboring subunits (**k**), with the unique closed state-stabilizing hydrogen bonds, which were not present in the open state, indicated by dashed lines and residues contributing to their formation shown as sticks. **l-m** Close-up views of the TRPM7-N1098Q$_{open}$ channel intracellular entrance (**l**) and S6-TRP helix regions in the neighboring subunits (**m**), with the unique open state-stabilizing hydrogen bonds indicated by dashed lines.

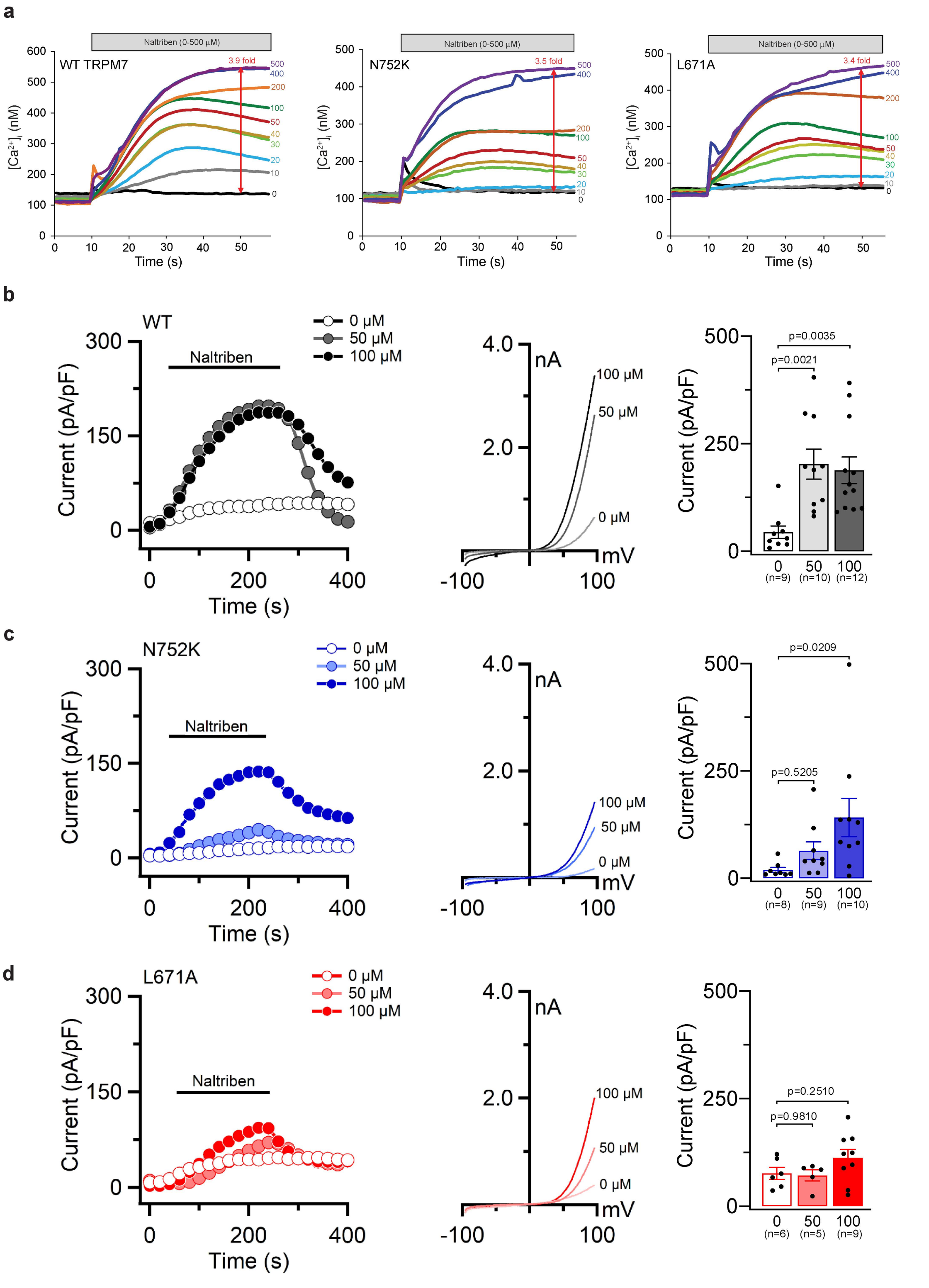

Supplementary Fig. 7 | Activation of wild-type and mutant TRPM7 currents by naltriben. a Representative measurements of NTB-stimulated Ca^{2+} influx in HEK 293T cells expressing wild-type (WT) and mutant TRPM7 channels. **b-d** Whole-cell currents were measured in HEK 293T cells expressing WT (**b**), N752K (**c**) and L671A (**d**) variants of TRPM7. *Left panels* show representative current amplitudes measured at +80 mV using the standard external solution without and with 50 μM or 100 μM NTB as indicated by the black bars. *Middle panels* show representative current-voltage (I–V) relationships obtained at 230 s in the *left panels*. *Right panels* show current amplitudes at +80 mV (mean ± SEM) obtained at 230 s as exemplified in the *left panels*. *n*, number of cells measured. Statistical comparisons were made using ordinary one-way ANOVA. Significance was accepted at $p \leq 0.05$.

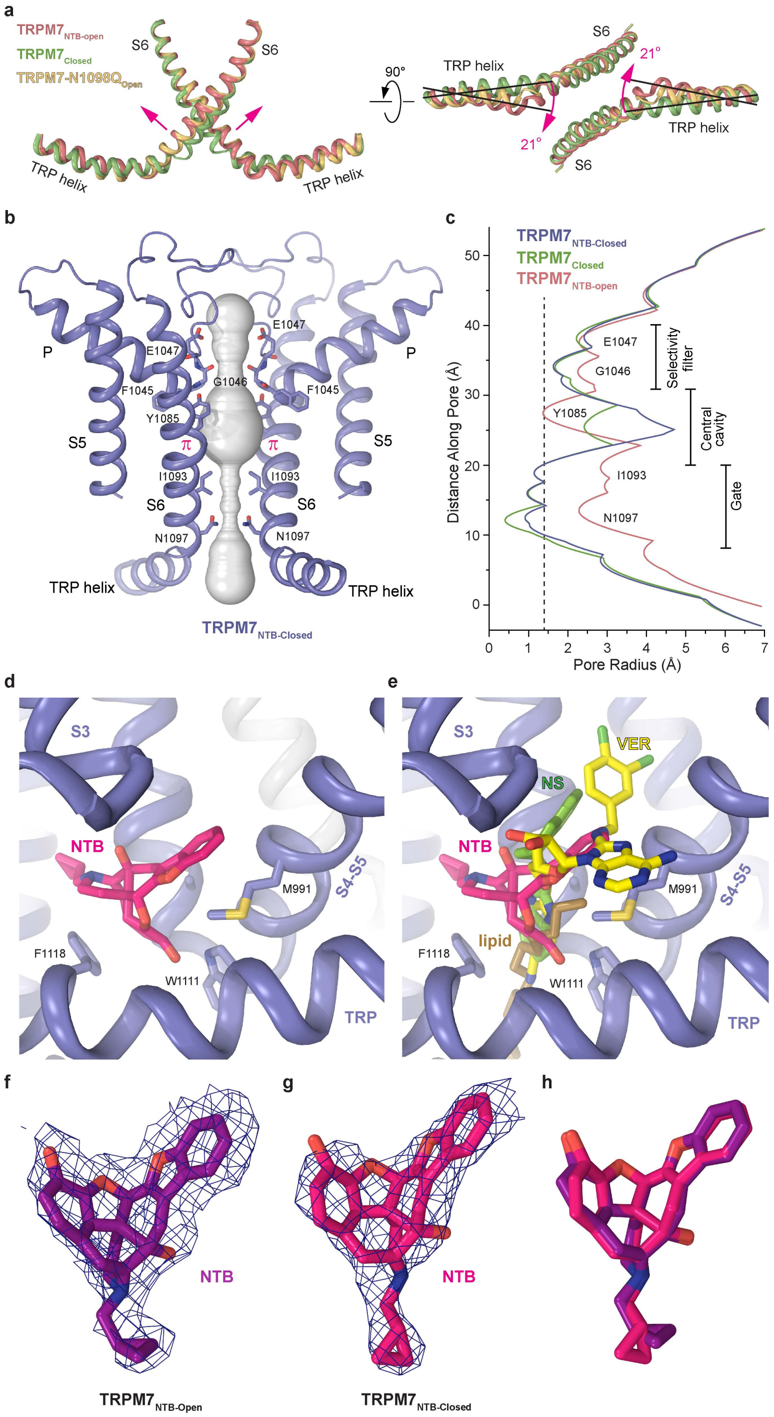

Supplementary Fig. 8 | Conformational changes in the pore domain of TRPM7$_{\text{NTB-open}}$ and naltriben binding to the vanilloid-like site in TRPM7$_{\text{NTB-closed}}$. **a** Superposition of S6 and TRP helices for two diagonal subunits in TRPM7$_{\text{NTB-open}}$ (red), TRPM7$_{\text{Closed}}$ (green) and TRPM7-N1098Q$_{\text{open}}$ (yellow), with the other two subunits removed for clarity. Pink arrows indicate domain movements between TRPM7$_{\text{Closed}}$ and TRPM7$_{\text{NTB-open}}$. **b** Pore-forming domain in TRPM7$_{\text{NTB-Closed}}$ with the residues contributing to pore lining shown as sticks. Only two of four subunits are shown, with the front and back subunits omitted for clarity. The pore profile is shown as a space-filling model (grey). The π-bulge in the middle of S6 is labeled. **c** Pore radius for TRPM7$_{\text{NTB-Closed}}$ (purple), TRPM7$_{\text{Closed}}$ (green) and TRPM7$_{\text{NTB-open}}$ (red) calculated using HOLE. The vertical dashed line denotes the radius of a water molecule, 1.4 Å. **d** Close-up view of the vanilloid-like site in TRPM7$_{\text{NTB-Closed}}$, with the molecule of NTB shown in sticks (pink). **e** The same region of TRPM7$_{\text{NTB-Closed}}$ as in (**d**), with the molecules of VER155008 from TRPM7$_{\text{VER-Closed}}$ (yellow), NS8593 from TRPM7$_{\text{NS-Closed}}$ (green) and the lipid (brown) from TRPM7$_{\text{Closed}}$ superposed. **f-g** Representative densities (blue mesh) for naltriben (NTB) in TRPM7$_{\text{NTB-Open}}$ (**f**) and TRPM7$_{\text{NTB-Closed}}$ (**g**). **h** Superposed NTB molecules from TRPM7$_{\text{NTB-Open}}$ (purple) and TRPM7$_{\text{NTB-Closed}}$ (pink) shown in sticks.

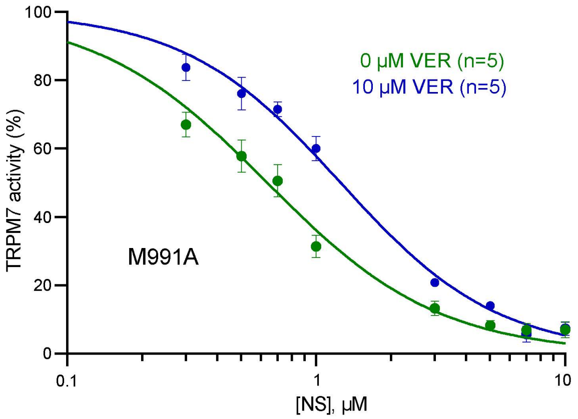

Supplementary Fig. 9 | Competition of the NS8593 and VER155009 inhibitors for the same binding site. Concentration dependencies of NS8593 inhibition for TRPM7-M991A in the absence and presence of 10 µM VER155009, assessed using the Ca^{2+} influx assay. Curves through the points (mean ± SEM) are logistic equation fits. *n,* the number of independent measurements. The values of IC_{50} and n_{Hill} are provided in **Supplementary Table 4**. Source data are provided. Note: the control data for TRPM7-M991A (without VER155008) were taken from experiments illustrated in **Fig. 6g**.

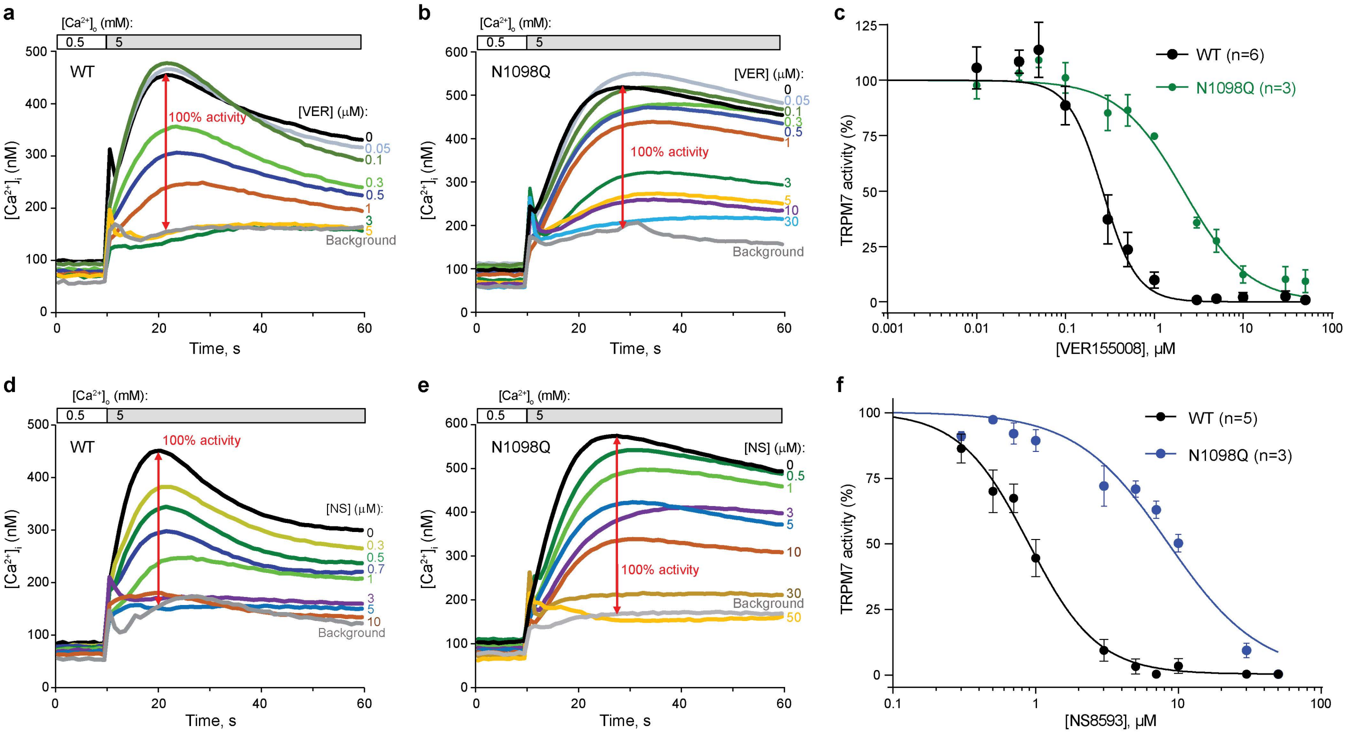

Supplementary Fig. 10 | Inhibition of wild-type and N1098Q TRPM7 by VER155008 and NS8593. A-b Representative Ca^{2+} uptake traces for wild-type (WT) (**a**) and mutant N1098Q (**b**) TRPM7 at different concentrations of VER155008. **c** Concentration-dependencies of WT and N1098Q TRPM7 inhibition by VER155008 measured using traces illustrated in (**a-b**). Curves through the points (mean ± SEM) are logistic equation fits, with the values of IC_{50} and n_{Hill} provided in **Supplementary Table 4**. *n*, the number of independent measurements. Source data are provided. **d-e** Representative Ca^{2+} uptake traces for WT (**d**) and N1098Q mutant (**e**) TRPM7 at different concentrations of NS8593. **f** Concentration-dependencies of WT and N1098Q TRPM7 inhibition by NS8593 measured using traces illustrated in (**d-e**). Curves through the points (mean ± S.E.M.) are logistic equation fits, with the values of IC_{50} and n_{Hill} provided in **Supplementary Table 4**. n, the number of independent measurements. Source data are provided.

Supplementary Table 1 | Cryo-EM data collection, refinement, and validation statistics.

**Structure**	**TRPM7$_{closed}$**	**TRPM7**	**TRPM7-N1098Q$_{open}$**	**TRPM7$_{NTB-open}$**	**TRPM7$_{NTB-closed}$**
Preparation	MSP2N2	GDN	GDN	MSP2N2	MSP2N2
Ligand	apo	apo	apo	Naltriben	Naltriben
EMDB accession code	EMDB-40496	EMDB-40497	EMDB-40498	EMDB-40499	EMDB-40500
PDB accession code	8SI2	8SI3	8SI4	8SI5	8SI6
**Data collection**					
Magnification	105,000x	105,000x	105,000x	105,000x	105,000x
Voltage (kV)	300	300	300	300	300
Electron exposure (e-/Å2)	58	58	58	58	58
Defocus range (μm)	–0.5 to –1.5	–0.5 to –1.5	–0.5 to –1.5	–0.5 to –1.5	–0.5 to –1.5
Reported pixel size (Å)	0.83	0.83	0.83	0.83	0.83
Calibrated pixel size (Å)	0.83	0.83	0.83	0.83	0.83
Exposures (no.)	5,087	9,822	5,521	5,738	5,738
**Processing**					
Platform software for particle picking	CryoSPARC v3.3.2	CryoSPARC v3.3.2	CryoSPARC v3.3.2	CryoSPARC v3.3.2	CryoSPARC v3.3.2
Motion correction	MotionCor2	Patch Motion	MotionCor2	MotionCor2	MotionCor2
CTF estimation	Patch CTF	Patch CTF	Patch CTF	Patch CTF	Patch CTF
Software for 2D/3D class. & refinements	Relion v4.0, Cryosparc v3.3.2	Cryosparc v3.3.2	Relion v4.0, Cryosparc v3.3.2	Relion v4.0, Cryosparc v3.3.2	Relion v4.0, Cryosparc v3.3.2
Symmetry imposed	C4	C4	C4	C4	C4
Initial particle images (no.)	1,336,887	3,033,187	1,597,469	1,408,817	1,408,817
Final particle images (no.)	178,432	122,316	157,446	134,039	96,984
Map resolution (Å) FSC 0.143	2.19	2.61	2.46	2.17	2.44
Map resolution range (Å) min/median/max	1.92/3.39/33.27	1.80/4.56/34.78	1.81/5.61/37.99	1.90/3.23/34.27	2.13/4.31/36.47
**Refinement**					
Initial models used (PDB code)	5zx5	This study	This study	This study	This study
Model resolution (Å) FSC threshold	2.19	2.61	2.46	2.17	2.44
Map sharpening *B* factor ($Å^2$)	–66	–81	–67	–60	–73
Model composition					
Non-hydrogen atoms	39,022	38,613	38,997	38,949	38,649
Protein residues	4,420	4,420	4,420	4,288	4,428
Ligand atoms	3,310	2,901	3,281	3,493	2,865
R.m.s. deviations					
Bond lengths (Å)	0.009	0.006	0.010	0.011	0.008
Bond angles (°)	1.004	1.135	1.354	1.179	0.959
Validation					
CC (across whole map volume)	0.6012	0.5777	0.5840	0.5906	0.3510
CC (only across atoms in the model)	0.6991	0.7039	0.6829	0.7493	0.7285
Clashscore, all atoms	2.35	2.78	2.97	2.55	1.75
Poor rotamers (%)	0.20	0.41	0.82	0.10	0.00
Ramachandran plot					
Favored (%)	90.90	90.63	87.90	91.96	91.77
Allowed (%)	8.83	8.74	11.37	7.86	7.51
Disallowed (%)	0.27	0.64	0.73	0.18	0.73

Supplementary Table 1 | Cryo-EM data collection, refinement, and validation statistics (continued).

**Structure**	**TRPM7$_{VER\text{-}Closed}$**	**TRPM7-N1098Q$_{VER\text{-}Closed}$**	**TRPM7-N1098Q$_{NS\text{-}Closed}$**	**TRPM7$_{closed}$ MHR1-3**
Preparation	GDN	GDN	GDN	MSP2N2
Ligand	VER155008	VER155008	NS8593	apo
EMDB accession code	EMDB-40501	EMDB-40502	EMDB-40504	EMDB-40505
PDB accession code	8SI7	8SI8	8SIA	8SIB
**Data collection**				
Magnification	105,000x	105,000x	105,000x	105,000x
Voltage (kV)	300	300	300	300
Electron exposure (e-/Å2)	58	58	60	58
Defocus range (μm)	–0.5 to –1.5	–0.5 to –1.5	–0.5 to –1.5	–0.5 to –1.5
Reported pixel size (Å)	0.83	0.83	0.428	0.83
Calibrated pixel size (Å)	0.83	0.83	0.81	0.83
Exposures (no.)	5,989	4,685	7,659	5,087
**Processing**				
Platform software for particle picking	CryoSPARC v3.3.2	CryoSPARC v3.3.2	CryoSPARC v3.3.2	CryoSPARC v3.3.2
Motion correction	Patch Motion Correction	MotionCor2	MotionCor2	MotionCor2
CTF estimation	Patch CTF	Patch CTF	Patch CTF	Patch CTF
Software for 2D/3D class. & refinements	Cryosparc v3.3.2	Relion v4.0, Cryosparc v3.3.2	Relion v4.0, Cryosparc v3.3.2	Relion v4.0, Cryosparc v3.3.2
Symmetry imposed	C4	C4	C4	C1
Initial particle images (no.)	1,425,193	1,083,549	1,380,320	1,336,887
Final particle images (no.)	197,764	93,797	139,500	325,415
Map resolution (Å) FSC 0.143	2.59	2.99	2.91	2.62
Map resolution range (Å) min/median/max	2.15/5.45/39.61	1.78/6.10/47.22	2.58/6.27/45.38	2.34/3.37/35.65
**Refinement**				
Initial models used (PDB code)	This study	This study	This study	This study
Model resolution (Å) FSC threshold	2.59	2.99	2.91	2.62
Map sharpening *B* factor ($Å^2$)	–79	–118	–101	–102
Model composition				
Non-hydrogen atoms	38,761	38,609	38,537	3,544
Protein residues	4,420	4,420	4,420	454
Ligand atoms	3,049	2,893	2,821	0
R.m.s. deviations				
Bond lengths (Å)	0.011	0.010	0.009	0.006
Bond angles (°)	1.082	1.113	1.402	1.214
Validation				
CC (across whole map volume)	0.6004	0.6737	0.6267	0.7279
CC (only across atoms in the model)	0.7259	0.7419	0.6625	0.8001
Clashscore, all atoms	2.83	3.34	3.52	4.20
Poor rotamers (%)	0.10	0.41	0.72	0.78
Ramachandran plot				
Favored (%)	90.10	86.60	84.96	85.78
Allowed (%)	9.17	12.67	14.49	12.89
Disallowed (%)	0.73	0.73	0.55	1.33

Supplementary Table 2 | Summary of MD simulations. The average root-mean-square deviation (r.m.s.d) values with respect to initial structures were calculated for all protein Cα atoms (residues 843 to 1147), S1-S6 and TRP helices (residues 853 to 875, 885 to 905, 914 to 943, 958 to 981, 986 to 1022, and 1071 to 1123), and the channel pore only (residues and 1085-1098 and 1045 to 1057).

System	Simulation length, ns	Number of atoms	Equilibrated box size, Å3	Cα RMSD, Å2		
				Entire model	S1-S6 and TRP helices	Channel pore
Simulations with no applied voltage						
$TRPM7_{Closed}$-rep1	1050	178521	134×137×94	1.94 ± 0.18	1.47 ± 0.15	1.08 ± 0.12
$TRPM7_{Closed}$-rep2	1020	178680	138×135×93	1.77 ± 0.27	1.31 ± 0.15	0.97 ± 0.14
$TRPM7_{NTB\text{-}Open}$	808	178443	137×136×93	1.53 ± 0.21	0.77 ± 0.06	0.63 ± 0.06
TRPM7-$N1098Q_{Open}$-rep1	1065	178458	135×135×95	2.01 ± 0.29	1.13 ± 0.09	0.61 ± 0.07
TRPM7-N1098QOpen-rep2	1078	178416	135×137×94	2.17 ± 0.33	1.17 ± 0.08	0.61 ± 0.06
Simulations under 600 mV voltage						
TRPM7-$N1098Q_{Open}$-rep1v	400	178353	131×137×96	2.20 ± 0.62	1.58 ± 0.41	0.87 ± 0.28
TRPM7-$N1098Q_{Open}$-rep2v	300	178404	132×136×96	2.66 ± 0.62	1.50 ± 0.38	0.58 ± 0.07
$TRPM7_{NTB\text{-}Open}$-rep1v	425	178365	135×133×96	1.37 ± 0.17	0.78 ± 0.07	0.76 ± 0.09
$TRPM7_{NTB\text{-}Open}$-rep2v	400	178365	135×133×96	1.45 ± 0.26	0.81 ± 0.07	0.75 ± 0.11

Supplementary Table 3 | Water permeation and ion conductance in MD simulated structures. Water permeation and ion conductance were computed using production trajectories of all systems as described in Methods. For the systems with two replicas, the values reported are averages over the two simulations.

System	Total simulation length, ns	Average water permeation per ns	Average ion conductance, pS
Simulations with no applied voltage			
$TRPM7_{Closed}$-rep1 (2 replicas)	2070	0.13 ± 0.22	
TRPM7-$N1098Q_{open}$ (2 replicas)	2143	9.45 ± 1.54	
$TRPM7_{NTB\text{-}open}$	808	7.58 ± 0.53	
Simulations under 600 mV voltage			
TRPM7-$N1098Q_{open}$ (2 replicas)	700	16.17 ± 2.29	16.8 ± 7.21
$TRPM7_{NTB\text{-}open}$ (2 replicas)	825	14.58 ± 1.36	3.69 ± 0.18

Supplementary Table 4 | *EC_{50}* values for TRPM7 activation by naltriben and *IC_{50}* values for TRPM7 inhibition by VER155008 and NS8593. Data are presented as the mean ± SEM.

TRPM7 variant	Ligand	Action	*EC_{50}* (μM)	Hill coefficient	n, independent measurements
WT	Naltriben	agonist	65.8 ± 12.36	1.23 ± 0.25	6
N752K	Naltriben	agonist	191.40 ± 17.96	1.91 ± 0.24	3
L671A	Naltriben	agonist	144.5 ± 22.91	1.68 ± 0.32	3
N752K/ L671A	Naltriben	agonist	230.1 ± 48.93	2.82 ± 1.38	4
S744A	Naltriben	agonist	71.8 ± 9.70	1.48 ± 0.25	4
M741A	Naltriben	agonist	85.0 ± 6.51	1.37 ± 0.11	4
D670A	Naltriben	agonist	117.3 ± 32.36	1.17 ± 0.24	3
**TRPM7 variant**	**Ligand(s)**	**Action**	***IC_{50}* (μM)**	**Hill coefficient**	**n, independent measurements**
WT	VER155008	antagonist	0.23 ± 0.03	–2.07 ± 0.41	6
N1098Q	VER155008	antagonist	1.71 ± 0.25	–1.32 ± 0.20	3
A981L	VER155008	antagonist	~25		3
W1111A	VER155008	antagonist	~10		3
WT	NS8593	antagonist	0.91 ± 0.08	–1.73 ± 0.26	5
N1098Q	NS8593	antagonist	20.47 ± 10.7	–0.93 ± 0.20	3
A981L	NS8593	antagonist	11.60 ± 2.75	–1.61 ± 0.45	3
A981V	NS8593	antagonist	21.91 ± 12.27	–1.45 ± 0.50	3
W1111A	NS8593	antagonist	5.14 ± 0.45	–1.54 ± 0.24	4
M991A	NS8593	antagonist	0.59 ± 0.05	–1.24 ± 0.15	5
M991A	NS8593 (+10 μM VER155008)	antagonist	1.28 ± 0.09	–1.43 ± 0.11	5

Reference

80. Henderson, R. et al. Outcome of the first electron microscopy validation task force meeting. Structure 20, 205–214 (2012).
