# Supplementary material for: Structural mechanisms of TRPM7 activation and inhibition
Source: Nat Commun. 2023 May 8;14:2639. doi: 10.1038/s41467-023-38362-3 (PMC10167348; doi:10.1038/s41467-023-38362-3)
Supplement: Supplementary file 3 — Description of Additional Supplementary Files [file 41467_2023_38362_MOESM3_ESM.pdf]

Description of Additional Supplementary Files

File Name: Supplementary Movie 1

Description: Permeation of Na^+ ions through the TRPM7-N1098Q_{open} channel in the absence of applied voltage. Two Na^+ ions permeating the lower gate are shown as orange and purple spheres. Non-permeating Na^+ ions are shown as grey spheres. The simulation was started with no ions in the pore and a Na+ ion (shown in orange) placed below the gate entrance. The protein is shown in yellow ribbon representation. Only the pore forming regions of two protein subunits are shown, with the front and back subunits omitted for clarity. Pore lining residues F1045, G1046, E1047, Y1085, I1093, and N1097 are shown as sticks. Water molecules are shown as transparent red and white balls and sticks. Lipids are shown as white sticks. The length of the shown trajectory is 4.8 ns.

File Name: Supplementary Movie 2

Description: Permeation of K^+ ions through the TRPM7-N1098Q_{open} channel under the applied voltage of 600 mV. The K^+ ions permeating the lower gate and/or the Y1085 level are shown as orange, purple, blue, and green spheres. Non-permeating K^+ ions are shown as grey spheres. The protein is shown in yellow ribbon representation. Only the pore forming regions of two protein subunits are shown, with the front and back subunits omitted for clarity. Pore lining residues F1045, G1046, E1047, Y1085, I1093, and N1097 are shown as sticks. Water molecules are shown as transparent red and white balls and sticks. Lipids are shown as white sticks. The length of the shown trajectory is 45 ns.

File Name: Supplementary Movie 3

Description: Structural rearrangements in TRPM7 caused by the N1098 mutation or binding of the agonist naltriben.
